# Antibiotic-potentiation activities of four Cameroonian dietary plants against multidrug-resistant Gram-negative bacteria expressing efflux pumps

**DOI:** 10.1186/1472-6882-14-258

**Published:** 2014-07-21

**Authors:** Francesco K Touani, Armel J Seukep, Doriane E Djeussi, Aimé G Fankam, Jaurès A K Noumedem, Victor Kuete

**Affiliations:** 1 Department of Biochemistry, Faculty of Science, University of Dschang, P.O. Box 67, Dschang, Cameroon

**Keywords:** Cameroonian dietary plants, Potentiation, Gram–negative bacteria, Multidrug resistant, Efflux pumps

## Abstract

**Background:**

The continuous spread of multidrug-resistant (MDR) bacteria, partially due to efflux pumps drastically reduced the efficacy of the antibiotic armory, increasing the frequency of therapeutic failure. The search for new compounds to potentiate the efficacy of commonly used antibiotics is therefore important. The present study was designed to evaluate the ability of the methanol extracts of four Cameroonian dietary plants (*Capsicum frutescens* L. var. *facilulatum*, *Brassica oleacera* L. var. *italica*, *Brassica oleacera* L. var. *butyris* and *Basilicum polystachyon* (L.) Moench.) to improve the activity of commonly used antibiotics against MDR Gram-negative bacteria expressing active efflux pumps.

**Methods:**

The qualitative phytochemical screening of the plant extracts was performed using standard methods whilst the antibacterial activity was performed by broth micro-dilution method.

**Results:**

All the studied plant extracts revealed the presence of alkaloids, phenols, flavonoids, triterpenes and sterols. The minimal inhibitory concentrations (MIC) of the studied extracts ranged from 256-1024 μg/mL. *Capsicum frutescens* var. *facilulatum* extract displayed the largest spectrum of activity (73%) against the tested bacterial strains whilst the lower MIC value (256 μg/mL) was recorded with *Basilicum polystachyon* against *E. aerogenes* ATCC 13048 and *P. stuartii* ATCC 29916. In the presence of PAβN, the spectrum of activity of *Brassica oleacera* var. *italica* extract against bacteria strains increased (75%). The extracts from *Brassica oleacera* var. *butyris, Brassica oleacera* var. *italica, Capsicum frutescens* var. *facilulatum* and *Basilicum polystachyon* showed synergistic effects (FIC ≤ 0.5) against the studied bacteria, with an average of 75.3% of the tested antibiotics.

**Conclusion:**

These results provide promising information for the potential use of the tested plants alone or in combination with some commonly used antibiotics in the fight against MDR Gram-negative bacteria.

## Background

The spread of multidrug-resistant bacteria, partially due to the inappropriate use of common antibiotics, drastically reduced the efficacy of the antibiotic armory, increasing the frequency of therapeutic failure. The over-expression of efflux pumps is the main resistance mechanism observed in many bacteria [1]. In Gram-negative bacteria, many of these efflux pumps belong to the resistance-nodulation-cell division (RND), family of tripartite efflux pumps [2]. In the fight against microbial infections including those due to MDR bacteria, investigations are being carried out to discover new effective, none or less-toxic and available antibacterial drugs. Many scientist are also investigating synergistic compounds to potentiate the activity of the commonly used antibiotics [3]. The present work was designed to evaluate the *in vitro* ability of some edible plants namely *Capsicum frutescens* L. var. *facilulatum* (Solanaceae) or ‘chili pepper’, *Brassica oleacera* L. var. *italica* commonly known as ‘Broccoli’ and *Brassica oleacera* L. var. *butyris* (Brassicaceae) or ‘Cauliflower’; and *Basilicum polystachyon* (L.) Moench. (Lamiaceae) or ‘Musk Basil’ to potentiate the effect of common antibiotics against Gram-negative MDR phenotypes.

## Methods

### Plant material and extraction

The plants used in this study were collected in Douala (Littoral Region of Cameroon) in January 2013. The plants were further identified at the National Herbarium (Yaoundé, Cameroon) where voucher specimens were deposited under a reference number (Table 1). Air dried and powdered sample (0.1 g) of each plant was extracted by maceration with methanol (0.3 L) for 48 h at room temperature (25°C). After filtration using Whatman No. 1 filter paper, the filtrate of each plant was concentrated under reduced pressure in a rotary evaporator, and dried at room temperature to give the crude extract. The extraction yield was calculated (Table 2). These extracts were then stored at 4°C until further use.

**Table 1:** Information on plants used in this study

**Plants samples and herbarium voucher number**^**a**^	**Parts used**	**Popular names**	**Traditional used**	**Known antimicrobial activities of plants**
***Capsicum frutescens *L. var. *facilulatum *(Solanaceae)** 43079/HNC	Fruits	Green pepper	Antimitogenic [4], allergy, cancer and viral infection [5]	Antibacterial activities of aqueous and methanolic extracts against Sa, St, Vc [6,7], antifungal activities of lectin against Af, [8]; antifungal activities of saponin CAY-1 against Ca, *Aspergillus Spp* and dermatophytes Tm, Tr et Mc [9]
***Brassica oleacera *L. var. *italica *(Brassicaceae)** 25686/SFR Cam	Leaves	*Brocoli*	Oxydative stress, cytotoxic [10]	Antibacterial activities of ethanolic extractsagainst Sa, Bc, Pa [11]. Antifungal activities against Sc, Te, Hm, Pm [12].
***Brassica oleacera *L. var. *butyris *(Brassicaceae)** 25686/SFR Cam	Leaves	Flower cabbage	Cytotoxic effect, antiproliférative, Oxydative stress [13].	Antibacterial activities of sulfur compounds MMTSO, AITC, MMTSO_2_ against Pp, Lm, Lp, Lb Lm Sa, Ea, Ec, Bs, St and antifungal against strains Sc, Te, Hm, Pm [12].
***Basilicum polystachyon *(L.) Moench. (Lamiaceae)** 38650/HNC	Leaves	Cotimandjo (Cameroon)	Infectious diseases, gastroenteritis [14].	Strong activities of acidic extracts against Gram (+), but less activities against Gram;. Strong antifungal activities of ethanolic and methanolic extracts against An [15].

Af, Fm Ca, Tm, Tr, Tt, Mc, Sa, Bc, Ec, Pa, Sc, Te Hm, Pm, Pp, Lm, Lp, Lb, Lm, Bs, Ea, St, Te, Hm, An, Kp, Ec, Sm, Vc who are respectively : *Aspergillus flavus, Fusarium moniliforme, Candidat albicans, trichophyton mentagrophytes, T. rubum, T.tonsuraus Microsporum canis, Staphylococcus aureus*, *Bacillus cereus, Escherichia coli*, *Pseudomonas aeroginosa, Saccharomyces cerevisiae, Torulopsis etchellsii, Hansenula mrakii, Pichia membranefaciens, Pediococcus pentosaceus, Leuconostoc mesenteroides, Lactobacillus plantarum , Lactobacillus brevis, Listeria monocytogenes, Bacillus subtilis, Enterobacter aerogenes, Salmonella. Typhimurium, Torulopsis etchellsii, Hansenula mrakii, Aspergillus niger, Klebsiella pneumoniae* CI, *Enterobacter cloacae* CI, CIv *Vibrio cholerae MMTSO*: Méthylmethanethiosulfinate, *AITC*: Allyisothyocyanate, *MMTS0*_*2*_: Méthylmethanethiosulfonat. *SRFC*: Company of Forest Reserve of Cameroon; *HNC*: Cameroon National Herbarium.

**Table 2:** Extraction yields and phytochemical composition of the studied plants

**Extract**	***Capsicum frutescens***	***Brassica oleacera *** **var. *butyris***	***Brassica oleacera *** **var. *italica***	***Basilicum polystachyon***
Yield* (%)	**7.22%**	**12.18%**	**7.31%**	**8.61%**
Physical aspect	Oily brown and viscous	Oily brown and viscous	Oily brown and viscous	Compact
Alkaloids	+	+	+	+
Anthocyanins	-	-	-	-
Anthraquinones	-	-	-	-
Flavonoids	+	+	+	+
Phenols	+	+	+	+
Coumarins	-	-	-	+
Tannins	-	+	+	-
Triterpenes	+	+	+	+
Sterols	+	+	+	+
Saponins	-	+	-	+

(+): Present; (-): Absent; *yield calculated as the ratio of the mass of the obtained methanol extract/mass of the plant powder.

### Preliminary phytochemical screenings

The secondary metabolite classes such as alkaloids, anthocyanins, anthraquinones, flavonoids, phenols, saponins, tannins, sterols and triterpenes were screened according to the standard phytochemical methods described by Harbone [16].

### Bacteria strains and culture media

The studied microorganisms included both reference (from the American Type Culture Collection, ATCC) and clinical (Laboratory collection) strains of *Escherichia coli*, *Enterobacter aerogenes, Providencia stuartii, Pseudomonas aeruginosa* and *Klebsiella pneumoniae* (Table 3). They were maintained at 4°C and sub-cultured on a fresh appropriate Mueller Hinton Agar (MHA) for 24 h before any antibacterial test. The Mueller Hinton Broth (MHB) was used for all antibacterial assays.

**Table 3:** Bacterial strains and features

**Bacteria and strains**		**Features**	**References**
***Escherichia coli***	ATCC 8739	References strains	
	ATCC 10536	References strains	
	AG100 Atet	AG 100 sur-expressing *AcrAB* pumps, contaning TET^R^ gène *acrF*	[14]
	AG100	Wild-typeE. ColiK-12	[15]
	AG102	AG100 Sur-exprissing*AcrAB* pumps.	[17]
	MC4100	*Wild typeE. coli*	
***Enterobacter aerogenes***	ATCC 13048	References strains	
	EA27	Clinical MDR isolate exhibiting energy-dependent norfloxacin and chloramphenicol efflux with KAN^R^ AMP^R^ NAL^R^ STR^R^ TET^R^	[18]
	EA-3	Clinical MDR isolate CHL^R^, NOR^R^, OFX^R^, SPX^R^, MOX^R^, CFT^R^, ATM^R^, FEP^R^	[18]
	EA 289	KAN sensitive derivative d’EA27	[18]
	EA 294	EA289 sur-expressing AcrA pumps Exhibiting KAN^R^	[18]
	EA 298	EA289 TolC KAN^R^	[18]
	CM64	CHL^R^resistant variant obtained from ATCC13048 over-expressing the AcrAB pump	[18]
***Klebsiella pneumoniae***	ATCC 11296	References strains	
	K-2	Clinical MDR isolate exhibiting energy-dependent norfloxacin and chloramphenicol efflux with KAN^R^ AMP^R^ NAL^R^ STR^R^ TET^R^	Laboratory collection of UNR-MD1, University of Marseille, France
	K-24	AcrAB-Tolc	
	KP 55	Clinical isolate MDR, TET^R^, AMP^R^, ATM^R^, CEF^R^	[17]
	KP 63	Clinical isolate du MDR, TET^R^, CHL^R^AMP^R^, ATM^R^	[17]
***Pseudomonas aeruginosa***	PA01	References strains	
	PA124	MDR Clinical isolate	[15]
***Providencia stuartii***	ATCC 29916	References strains	
	NAE16	MDR clinical isolate AcrAB-TolC	[15]

^a^AMP, ATM^R^, CEF^R^, CFT^R^, CHL^R^, FEP^R^, KAN^R^, MOX^R^, STR^R^, TET^R^. Resistance to ampicillin, aztreonam, cephalothin, cefadroxil, chloramphenicol, cefepime, kanamycin, moxalactam, streptomycin, and tetracycline; OMPF and *OMPC*: Outer Membran Protein F and C respectively. *AcrAB-Tol*: Efflux pump of type AcrAB associated to one porine of type TolC.

### Chemicals for antibacterial assays

Nine commonly used antibiotics including tetracycline (TET), cefepime (CEP), streptomycin (STR), ciprofloxacin (CIP), norfloxacin (NOR), chloramphenicol (CHL), ampicillin (AMP), erythromycin (ERY), kanamycin (KAN) (Sigma-Aldrich, St Quentin Fallavier, France) were used for potentiation assay. *p-*Iodonitrotetrazolium chloride 0.2% (INT) and phenylalanine arginine *β*-naphthylamide (PAβN) (Sigma-Aldrich) were used as bacterial growth indicator and efflux pumps inhibitor respectively. Dimethylsulfoxide 10% (DMSO) was used as solvent for all extracts.

### Bacterial susceptibility determinations

The minimal inhibitory concentrations (MIC) of the plant extracts against the studied bacteria were determined by rapid INT colorimetric assay [19,20]. Briefly, the test samples were first dissolved in DMSO/MHB. The solution obtained was then added to MHB in a 96-well microplate followed by a two fold serial dilution. One hundred microliters (100 μL) of inoculum (1.5 × 10^6^ CFU/mL) prepared in MHB was then added. The plates were covered with a sterile plate sealer, then agitated to mix the contents of the wells using a shaker and incubated at 37°C for 18 h. The final concentration ranges were 8-1024 μg/mL for plant extracts and 2-512 μg/mL for reference antibiotic chloramphenicol (CHL). Wells containing MHB (100 μL), 100 μL of inoculum and DMSO at a final concentration of 2.5% served as negative growth inhibition control. MIC was detected after 18 h of incubation at 37°C, following addition (40 μL) of 0.2 mg/mL INT and incubation at 37°C for 30 min. Viable bacteria reduced the yellow dye to pink. MIC was defined as the lowest sample concentration that prevented this change and exhibited complete inhibition of bacterial growth [21]. The minimal bactericidal concentrations (MBC) of the samples was determined by taking 50 μL of the suspensions from the wells which did not show any growth after incubation during MIC assays to a new 96-well microplate containing 150 μL of fresh broth per well. The plate was further re-incubated at 37°C for 48 hours the addition of INT. The MBC was defined as the lowest concentration of samples which completely inhibited the growth of bacteria. Samples were tested alone and in the presence of PAβN at 30 μg/mL final concentration [22].

To evaluate the potentiating effect of tested extracts, a preliminary combination at their sub-inhibitory concentrations (MIC/2, MIC/5, MIC/10 and MIC/20) with antibiotics was assessed against *P. aeruginosa* PA124 strain. The appropriate sub-inhibitory concentrations were then selected on the basis of their ability to improve the activity of the maximum antibiotic. These sub-inhibitory concentrations for selected extracts were further tested in combination with antibiotics against more MDR bacteria. The Fractional inhibitory concentration (FIC) of each combination was then calculated as the ratio of MIC of Antibiotic in combination versus MIC of Antibiotic alone [23,24].

## Results

### Phytochemical composition of the tested plant’s extracts

The results of the qualitative phytochemical analysis showed that each of the studied extract contained alkaloids, phenols, flavonoids, triterpenes and sterols. None of them contained anthocyanins and anthraquinones. Other phytochemical classes have been selectively detected as shown in Table 2.

### Antibacterial activity of the plant’s extracts

Bacterial strains and MDR isolates were tested for their susceptibility to plant extracts and chloramphenicol. The results summarized in Table 4 the selectivity of the extracts towards the tested bacteria, with MIC values ranging from 256 to 1024 μg/mL on the majority of the 22 tested microorganisms. *Capsicum frutescens* extract displayed the largest spectrum of activity, 73% (16/22) against the tested bacteria; followed by *Brassica oleacera* var. *italica,* 50% (11/22); *Basilicum polystachyon* 41% (9/22) and *Brassica oleacera* var. *butyris* 27% (6/22) extracts. The lowest MIC value (256 μg/mL) was recorded with *Basilicum polystachyon* extract against *P. stuartii* (ATCC 29916) and *E. aerogenes* (ATCC 13048). No significant MBC value was recorded.

**Table 4:** MIC and MBC of the tested plants extracts and CHL on the studied bacterial species

**Strains bacterial**		***Capsicum frutescens***		***Brassica oleacera *** **var. *varbutyris***		***Brassica oleacera *** **var. *italica***		***Basilicum polystachyon***		**Chloramphenicol**	
		**MIC**	**MBC**	**MIC**	**MBC**	**MIC**	**MBC**	**MIC**	**MBC**	**MIC**	**MBC**
***Escherichia coli***	ATCC 8739	-	-	1024	-	-	-	-	-	8	512
	ATCC 10536	512	-	-	-	1024	-	-	-	2	128
	AG100 *Atet*	512	-	1024	-	1024	-	512	1024	64	64
	AG100	-	-	-	-	-	-	-	-	16	128
	AG102	1024	-	-	-	1024	-	1024	-	8	-
	MC4100	1024	-	512	-	512	-	1024	-	128	128
***Enterobacter aerogenes***	ATCC 13048	1024	-	1024	-	1024	-	**256**	-	8	32
	EA27	-	-	-	-	-	-	-	-	256	NT
	EA-3	1024	-	-	-	1024	-	1024	-	-	-
	EA294	-	-	-	-	-	-	-	-	256	512
	EA298	512	-	-	-	-	-	-	-	4	16
	EA 289	1024	-	-	-	-	-	-	-	128	-
	CM64	1024	-	-	-	1024	-	-	-	128	-
***Klebsiella pneumoniae***	ATCC 11296	1024	-	1024	-	1024	-	-	-	8	512
	K-2	512	-	-	-	1024	-	512	-	64	NT
	K-24	1024	-	-	-	1024	-	1024	-	16	256
	KP 55	1024	-	-	-	-	-	512	-	32	-
	KP 63	512	-	-	-	-	-	-	-	128	NT
***Pseudomonas aeruginosa***	PA01	-	-	-	-	-	-	-	-	64	NT
	PA124	-	-	-	-	-	-	-	-	512	NT
***Providencia stuartii***	ATCC 29916	1024	-	1024	-	1024	-	**256**	-	4	32
	NAE16	1024	-	-	-	-	-	-	-	256	NT

*NT*: Not determined; -: superior to 1024 μL for extracts and superior to 512 μg/mL for antibiotics; *CHL*: Chloramphenicol; Values in Bold are the lowest MIC values for the plant extracts.

Eight (8) of the twenty two (22) studied MDR bacteria were also tested for their susceptibility to the plant extracts in the presence of PAβN (Table 5). The largest spectrum of activity was recorded with *B. oleacera* var*. butyris* extract against 75% (6/8) tested MDR bacteria. This efflux pumps inhibitor (EPI) also improved the activity of *C. frutescens* extract against *E. coli* (AG100), *K. pneumoniae* (KP53) and *E. aerogenes* (EA27) as well as that of *B. polystachyon* against *P. stuartii* (NAE16).

**Table 5:** Antibacterial activities of extracts alone and in the presence of PAβN

**Bacterial strains**	***Capsicum frutescens***	***Brassica oleacera *** **var. *butyris***	***Brassica oleacera *** **var** ***. italica***	***Basilicum polystachyon***	**CHL**	**PAβN**
AG100	1024 (256)	- (1024)	- (1024)	- (-)	16 (4)	>128
AG100 Atet	512 (512)	1024 (512)	1024 (1024)	- (-)	64(32)	>128
CM64	1024 (1024)	- (-)	1024 (512)	1024 (1024)	128 (64)	>128
EA27	- (512)	- (128)	- (512)	- (-)	256 (64)	>128
KP55	- (-)	- (1024)	- (1024)	- (-)	64(8)	>128
KP63	512 (256)	- (1024)	- (-)	- (-)	128(16)	>128
PA124	- (-)	- (-)	- (-)	- (-)	512(128)	>128
NAE16	- (-)	- (1024)	- (-)	- (1024)	256(64)	>128

( ): MIC value of extract in presence of PAβN; -: >1024 μg/mL for extracts and >512 μg/mL for antibiotic; *CHL*: Chloramphenicol.

### Antibacterial activity of extract-antibiotic combination

A preliminary assay against *P. aeruginosa* PA124 strain allowed selecting MIC/2 and MIC/5 as appropriate sub-inhibitory concentrations to be used on other bacteria (Table 6). Synergistic effects were observed with all the tested extracts. *Brassica oleacera* var*. italica* and *B. oleacera* var. *butyris* extracts potentiate (0.125 < FIC < 0.5 and 0.031 < FIC < 0.5 respectively) the effects of the majority of antibiotics on most of the tested MDR bacteria (Table 7). Extracts from *C. frutescens* and *B. polystachyon* showed synergistic effects with six of the nine studied antibiotics, with 0.125 < FIC < 0.5 and 0.25 < FIC < 0.5 respectively.

**Table 6:** **MICs of antibiotics in combination with plant extracts against *P. aeruginosa *** **PA124**

**Plants’ extracts**		**CEF**	**AMP**	**CIP**	**ERY**	**KAN**	**TET**	**STR**	**CHL**	**NOR**
** ATB ALONE**		- (-)	- (-)	**64**	**512**	**128**	**64**	**64**	**512**	**256**
***Capsicum frutescens***	MIC/2	- (-)	- (-)	**32 (0,5)**^**S**^	**256 (0,5)**^**S**^	128 (1)^1^	**32 (0,5)**^**S**^	256 (4)^1^	**256 (0,5)**^**S**^	**128 (0,5)**^**S**^
	MIC/5	- (-)	- (-)	**32 (0,5)**^**S**^	**256(0,5)**^**S**^	128 (1)^1^	64 (1)^1^	256 (4)^1^	**256 (0,5)**^**S**^	**128 (0,5)**^**S**^
	MIC/10	- (-)	- (-)	**32 (0,5)**^**S**^	**256 (0,5)**^**S**^	128 (1)^1^	64 (1)^1^	256 (4)^1^	**256 (0,5)**^**S**^	**128 (0,5)**^**S**^
	MIC/20	- (-)	- (-)	**64 (1)**^**1**^	**256 (0,5)**^**S**^	256 (2)^1^	64 (1)^1^	256(4)^1^	**256 (0,5)**^**S**^	**128 (0,5)**^**S**^
***Brassica oleacera *** **var. *butyris***	MIC/2	- (-)	- (-)	**32 (0,5)**^**S**^	**256 (0,5)**^**S**^	**16 (0,125)**^**S**^	**16 (0,25)**^**S**^	**32 (0,5)**^**S**^	**256 (0,5)**^**S**^	**128 (0,5)**^**S**^
	MIC/5	- (-)	- (-)	**32(0,5)**^**S**^	**256 (0,5)**^**S**^	**16 (0,125)**^**S**^	**16 (0,25)**^**S**^	**32 (0,5)**^**S**^	**256 (0,5)**^**S**^	**128 (0,5)**^**S**^
	MIC/10	- (-)	- (-)	**32(0,5)**^**S**^	**256 (0,5)**^**S**^	**16 (0,125)**^**S**^	**32 (0,25)**^**S**^	**32 (0,5)**^**S**^	**256 (0,5)**^**S**^	**128 (0,5)**^**S**^
	MIC/20	- (-)	- (-)	**32 (0,5)**^**S**^	**256 (0,5)**^**S**^	**32 (0,25)**^**S**^	**32 (0,25)**^**S**^	64 (1)^1^	**256 (0,5)**^**S**^	**128 (0,5)**^**S**^
***Brassica oleacera *** **var. *Italica***	MIC/2	- (-)	- (-)	**32 (0,5)**^**S**^	**256 (0,5)**^**S**^	128 (1)^1^	**32 (0,25)**^**S**^	**32 (0,5)**^**S**^	**256 (0,5)**^**S**^	**128 (0,5)**^**S**^
	MIC/5	- (-)	- (-)	64(1)^1^	**256 (0,5)**^**S**^	128 (1)^1^	**32 (0,25)**^**S**^	**32 (0,5)**^**S**^	**256 (0,5)**^**S**^	**128 (0,5)**^**S**^
	MIC/10	- (-)	- (-)	64 (1)^1^	**256 (0,5)**^**S**^	128 (1)^1^	**32 (0,25)**^**S**^	64 (1)^1^	512 (1)^1^	256 (1)^1^
	MIC/20	- (-)	- (-)	64(1)^1^	**256 (0,5)**^**S**^	128 (1)^1^	64 (1)^1^	64 (1)^1^	512 (1)^1^	256 (1)^1^
***Basilicum polystachyon***	MIC/2	- (-)	- (-)	**32 (0,5)**^**S**^	**128 (0,25)**^**S**^	256(2)^1^	64 (1)^1^	64(1)^1^	**256 (0,5)**^**S**^	256 (1)^1^
	MIC/5	- (-)	- (-)	**32 (0,5)**^**S**^	**256 (0,5)**^**S**^	256 (2)^1^	64 (1)^1^	64( 1)^1^	**256 (0,5)**^**S**^	256 (1)^1^
	MIC/10	- (-)	- (-)	64 (1)^1^	**256 (0,5)**^**S**^	256 (2)^1^	64 (1)^1^	64 (1)^1^	**256 (0,5)**^**S**^	256 (1)^1^
	MIC/20	- (-)	- (-)	64 (1)^1^	**256 (0,5)**^**S**^	256 (2)^1^	64 (1)^1^	64 (1)^1^	**256 (0,5)**^**S**^	256 (1)^1^

*s*: Synergy; *1*: Indifference; *A*: Antagonism; ( ): fractional inhibitory concentration or FIC; -: MIC > 512 μg/mL; *ATB*: Antibiotic; *CIP*: Ciprofloxacin, *NOR*: Norfloxacin, *CHL*: Chloramphenicol, *STR*: Streptomycin, *TET*: Tetracycline, *KAN*: Kanamycin, *ERY*: Erythromycin, *AMP*: Ampicillin and *CEF:* Cefepime; The values in bold represent the cases of synergy between extract and antibiotic.

**Table 7:** MIC of antibiotics in combination with plant at their MIC/2 and MIC/5 against selected MDR bacteria strains

**Antibiotics**	**Plant extracts and MIC**									
	**Bacterial strains**		***Capsicum frutescens***		***Brassica oleacera *** **var. *butyris***		***Brassica oleacera *** **var. *Italica***		***Basilicum polystachyon***	
		**MIC**	**MIC/2**	**MIC/5**	**MIC/2**	**MIC/5**	**MIC/2**	**MIC/5**	**MIC/2**	**MIC/5**
CEF	AG100	-	-	-	-	-	-	-	-	-
	EA27	256	-	-	-	-	**128 (0.5) **^**s**^	256 (1)^1^	256 (1)^1^	256 (1)^1^
	CM64	-	-	-	-	-	-	-	-	-
	KP55	-	-	-	-	-	-	-	-	-
	KP63	-	-	-	-	-	-	-	-	-
	NAE16	-	-	-	-	-	-	-	-	-
	PA124									
AMP	AG100	-	-	-	-	-	-	-	-	-
	EA27	-	-	-	-	-	-	-	-	-
	CM64	-	-	-	-	-	-	-	-	-
	KP55	-	-	-	-	-	-	-	-	-
	KP63	-	-	-	-	-	-	-	-	-
	NAE16	-	-	-	-	-	-	-	-	-
CIP	AG100	32	32 (1)^I^	64 (2)^1^	**8 (0.25)**^**S**^	**8 (0.25)**^**S**^	64 (2)^1^	64 (2)^I^	64 (2)^1^	128 (4)^1^
	EA27	16	32 (2)^1^	32 (2)^1^	**4 (0.25)**^**S**^	**4 (0.25)**^**S**^	**8 (0.5)**^**S**^	**8 (0.5)**^**S**^	128 (8)^A^	128 (8)^A^
	CM64	16	16 (1)^I^	16 (1)^I^	16 (1)^I^	16 (1)^I^	16 (1)^I^	16 (1)^I^	64 (4)^1^	128 (8)^A^
	KP55	16	**4 (0.25)**^**S**^	**8 (0.5)**^**S**^	**2 (0.125)**^**S**^	**4 (0.25)**^**S**^	**4 (0.25)**^**S**^	16 (1)^I^	**8 (0.5)**^**S**^	**8 (0.5)**^**S**^
	KP63	8*	**4 (0.5)**^**S**^	**4 (0.5)**^**S**^	1* (0.125)^**S**^	1* (0.125)^**S**^	**1 (0.125)**^**S**^	4 (0.25)^S^	16 (1)^I^	16 (1)^I^
	NAE16	8*	**2 (0.25)**^**S**^	**2 (0.25)**^**S**^	2* (0.25)^**S**^	2* (0.25)^**S**^	**2 (0.25)**^**S**^	2 (0.25)^S^	8* (1)^I^	8 (1)^I^
	PA124	64	**32 (0.5)**^**S**^	**32 (0.5)**^**S**^	**32 (0.5)**^**S**^	**32 (0.5)**^**S**^	**32 (0.5)**^**S**^	64 (1)^1^	**32 (0.5)**^**S**^	**32 (0.5)**^**S**^
ERY	AG100	32	**16 (0.5)**^**S**^	**16 (0.5)**^**S**^	**8 (0.25)**^**S**^	**8 (0.25)**^**S**^	**16 (0.5)**^**S**^	**16 (0.5)**^**S**^	64 (2)^1^	64 (2)^1^
	EA27	32	32 (1)^I^	32 (1)^I^	64 (2)^1^	64 (2)^1^	64 (2)^1^	64 (2)^1^	64 (2)^1^	64 (2)^1^
	CM64	32	64 (2)^1^	**16 (0.5)**^**S**^	32 (1)^I^	32 (1)^I^	32 (1)^I^	32 (1)^I^	64 (2)^1^	64 (2)^1^
	KP55	128	**128 (1)**^**I**^	**128 (1)**^**I**^	**64 (0.5)**^**S**^	**64 (0.5)**^**S**^	**64 (0.5)**^**S**^	128 (1)^I^	256 (2)^1^	256 (2)^1^
	KP63	128	**32 (0.25)**^**S**^	**64 (0.5)**^**S**^	**32 (0.25)**^**S**^	**64 (0.5)**^**S**^	**64 (0.5)**^**S**^	**64 (0.5)**^**S**^	256 (2)^1^	256 (2)^1^
	NAE16	128	**16 (0.125)**^**S**^	**16 (0.125)**^**S**^	**32 (0.25)**^**S**^	**64 (0.5)**^**S**^	128 (1)^I^	128 (1)^I^	256 (2)^1^	256 (2)^1^
	PA124	512	**256 (0.5)**^**S**^	**256 (0.5)**^**S**^	**256 0.5)**^**S**^	**256 (0.5)**^**S**^	256 (0.5)^S^	**256 (0.5)**^**S**^	**128 (0.25)**^**S**^	**256 0.5)**^**S**^
KAN	AG100	32	32 (1)^I^	64 (2)^1^	**16 (0.5)**^**S**^	**16 (0.5)**^**S**^	32 (1)^I^	32 (1)^I^	32 (1)^I^	32 (1)^I^
	EA27	32	**8 (0.25)**^**S**^	**8 (0.25)**^**S**^	**8 (0.25)**^**S**^	**8 (0.25)**^**S**^	16 (0.5)^S^	**16 (0.25)**^**S**^	64 (2)^1^	64 (2)^1^
	CM64	64	64 (1)^I^	64 (1)^I^	**16 (0.25)**^**S**^	**32 (0.5)**^**S**^	32 (0.5)^S^	**32 (0.5)**^**S**^	**32 (0.5)**^**S**^	**32 (0.5)**^**S**^
	KP55	64	**16 (0.25)**^**S**^	**16 (0.25)**^**S**^	**16 (0.25)**^**S**^	**16 (0.25)**^**S**^	16 (0.25)^S^	**16 (0.25)**^**S**^	**16 (0.25)**^**S**^	**16 (0.25)**^**S**^
	KP63	64	64 (1)^I^	64 (1)^I^	**16 (0.25)**^**S**^	**16 (0.25)**^**S**^	**16 (0.25)**^**S**^	**32 (0.5)**^**S**^	**32 (0.5)**^**S**^	**32 (0.5)**^**S**^
	NAE16	64	64 (1)^I^	64 (1)^I^	**32 (0.5)**^**S**^	**32 (0.5)**^**S**^	64 (1)^I^	64 (1)^I^	64 (1)^I^	64 (1)^I^
	PA124	128	128 (1)^I^	128 (1)^I^	**16 (0.125)**^**S**^	**16 (0.125)**^**S**^	128 (1)^I^	128 (1)^I^	256 (2)^1^	256 (2)^1^
TET	AG100	32	**8 (0.25)**^**S**^	**8 (0.25)**^**S**^	**16 (0.5)**^**S**^	**16 (0.5)**^**S**^	**4 (0.25)**^**S**^	**4 (0.125)**^**S**^	**16 (0.5)**^**S**^	32 (1)^I^
	EA27	128	**64 (0.5)**^**S**^	**64 (0.5)**^**S**^	**16 (0.125)**^**S**^	**16 (0.125)**^**S**^	**4 (0.031)**^**S**^	**32 (0.25)**^**S**^	**64 (0.5)**^**S**^	**64 (0.5)**^**S**^
	CM64	64	128 (2)^1^	128 (2)^1^	**4 (0.062)**^**S**^	**8 (0.125)**^**S**^	64 (1)^I^	64 (1)^I^	128 (2)^1^	256 (4)^1^
	KP55	16	**2 (0.125)**^**S**^	**4 (0.25)**^**S**^	**1 (0.062)**^**S**^	**1 (0.062)**^**S**^	**2 (0.125)**^**S**^	**2 (0.125)**^**S**^	16 (1)^I^	16 (1)^I^
	KP63	32	**8 (0.25)**^**S**^	**8 (0.25)**^**S**^	**8 (0.25)**^**S**^	**16 (0.5)**^**S**^	**16 (0.5)**^**S**^	**8 (0.25)**^**S**^	**16 (0.5)**^**S**^	**16 (0.5)**^**S**^
	NAE16	128	**64 (0.5)**^**S**^	**64 (0.5)**^**S**^	128 (1)^I^	128 (1)^I^	**64 (0.5)**^**S**^	**64 (0.5)**^**S**^	-	-
	PA124	64	**32 (0.5)**^**S**^	64 (1)^I^	**16 (0.25)**^**S**^	**16 (0.25)**^**S**^	**32 (0.5)**^**S**^	**32 (0.5)**^**S**^	64 (1)^I^	64 (1)^I^
STR	AG100	64	256 (4)^1^	256 (4)^1^	128 (1)^I^	128 (1)^I^	**64 (0.5)**^**S**^	128 (1)^I^	128 (1)^I^	128 (1)^I^
	EA27	8	32 (4)^1^	32 (4)^1^	4 (0.5)^S^	8 (1)^I^	**2 (0.25)**^**S**^	**2 (0.5)**^**S**^	8 (1)^I^	8 (1)^I^
	CM64	64	256 (4)^1^	256 (4)^1^	**8 (0.125)**^**S**^	**16 (0.5)**^**S**^	**16 (0.5)**^**S**^	**16 (0.5)**^**S**^	64 (1)^I^	64 (1)^I^
	KP55	16	32 (4)^1^	32 (4)^1^	16 (1)^I^	16 (1)^I^	16 (1)^I^	16 (1)^I^	16 (1)^I^	16 (1)^I^
	KP63	64	256 (4)^1^	256 (4)^1^	128 (1)^I^	128 (1)^I^	128 (1)^I^	128 (1)^I^	128 (1)^I^	128 (1)^I^
	NAE16	64	256 (4)^1^	256 (4)^1^	**64 (0.5)**^**S**^	**64 (0.5)**^**S**^	**64 (0.5)**^**S**^	**64 (0.5)**^**S**^	128 (1)^I^	128 (1)^I^
	PA124	64	256 (4)^1^	256 (4)^1^	**32 (0.5)**^**S**^	**32 (0.5)**^**S**^	**32 (0.5)**^**S**^	**32 (0.5)**^**S**^	64 (1)^I^	64 (1)^I^
CHL	AG100	16	**4 (0.25)**^**S**^	**4 (0.25)**^**S**^	**4 (0.25)**^**S**^	**4 (0.25)**^**S**^	16 (1)^I^	16 (1)^I^	16 (1)^I^	16 (1)^I^
	EA27	256	-	-	**64 (0.25)**^**S**^	**128 (0.5)**^**S**^	**32 (0.125)**^**S**^	**64 (0.25)**^**S**^	256 (1)^I^	256 (1)^I^
	CM64	128	**32 (0.25)**^**S**^	**32 (0.25)**^**S**^	**16 (0.125)**^**S**^	**16 (0.125)**^**S**^	**64 (0.5)**^**S**^	**64 (0.5)**^**S**^	256 (2)^1^	256 (2)^1^
	KP55	64	**32 (0.5)**^**S**^	**32 (0.5)**^**S**^	**16 (0.25)**^**S**^	**32 (0.5)**^**S**^	**32 (0.5)**^**S**^	**32 (0.5)**^**S**^	64(1)^1^	64 (1)^1^
	KP63	128	128 (1)^I^	128 (1)^I^	**64 (0.5)**^**S**^	**64 (0.5)**^**S**^	**32 (0.125)**^**S**^	**32 (0.5)**^**S**^	**64 (0.5)**^**S**^	**64 (0.5)**^**S**^
	NAE16	256	**16 (0.062)**^**S**^	**32 (0.125)**^**S**^	**8 (0.031)**^**S**^	**16 (0.062)**^**S**^	**32 (0.125)**^**S**^	**32 (0.125)**^**S**^	**128 (0.5)**^**S**^	**128 (0.5)**^**S**^
	PA124	512	**256 (0.5)**^**S**^	**256 (0.5)**^**S**^	**256 (0.5)**^**S**^	**256 (0.5)**^**S**^	**256 (0.5)**^**S**^	**256 (0.5)**^**S**^	**256 (0.5)**^**S**^	**256 (0.5)**^**S**^
NOR	AG100	16	16 (1)^I^	16 (1)^I^	**4 (0.25)**^**S**^	**8 (0.5)**^**S**^	**8 (0.5)**^**S**^	**8 (0.5)**^**S**^	16 (1)^I^	16 (1)^I^
	EA27	16	128 (4)^1^	128 (4)^1^	**8 (0.5)**^**S**^	**8 (0.5)**^**S**^	**2 (0.125)**^**S**^	**4 (0.25)**^**S**^	**8 (0.5)**^**S**^	16 (1)^I^
	CM64	128	256 (2)^1^	256 (2)^1^	**8 (0.0625)**^**S**^	**16 (0.125)**^**S**^	128 (1)^I^	128 (1)^I^	256 (2)^1^	256 (2)^1^
	KP55	128	**64 (0.5)**^**S**^	**64 (0.5)**^**S**^	**64 (0.5)**^**S**^	**64 (0.5)**^**S**^	**8 (0.0625)**^**S**^	**16 (0.125)**^**S**^	**64 (0.5)**^**S**^	128 (1)^I^
	KP63	8	32 (4)^1^	32 (4)^1^	**4 (0.25)**^**S**^	**4 (0.25)**^**S**^	8 (1)^I^	8 (1)^I^	8 (1)^I^	8 (1)^I^
	NAE16	32	**8 (0.25)**^**S**^	**16 (0.5)**^**S**^	**8 (0.25)**^**S**^	**8 (0.25)**^**S**^	**4 (0.125)**^**S**^	**4 (0.125)**^**S**^	**8 (0.25)**^**S**^	**8 (0.25)**^**S**^
	PA124	256	**128 (0.5)**^**S**^	**128 (0.5)**^**S**^	**128 (0.5)**^**S**^	**128 (0.5)**^**S**^	**128 (0.5)**^**S**^	**128 (0.5)**^**S**^	256 (1)^1^	256 (1)^1^

*s*: Synergy; *I*: Indifference; *A*: Antagonism; ( ): FIC values; -: MIC > 512 μg/mL or not determined FIC; *ATB*: Antibiotic; *CIP*: Ciprofloxacin, *NOR*: Norfloxacin, *CHL*: Chloramphenicol, *STR*: Streptomycin, *TET*: Tetracycline, *KAN*: Kanamycin, *ERY*: Erythromycin, *AMP*: Ampicillin and *CEF* Cefepime; The values in bold represent the cases of synergy between extract and antibiotic.

## Discussion

The Pharmacological potencies of plants’ secondary metabolites are well demonstrated. The qualitative phytochemical screening of the plant extracts showed the presence of several classes of secondary metabolites, such as alkaloids, flavonoids, phenols, triterpenes, sterols, saponins, tannins and coumarins. Several antibacterial activities associated to the presence of compounds belonging to these various classes were shown [25-27]. It should however be mentioned that the detection of an alleged bioactive class of secondary metabolite in a plant is not a guarantee for any biological property, as this will depend on the nature of the compounds as well as their concentrations and the possible interactions with other constituents [12]. The differences observed between the antibacterial activities of the extracts as observed in the present work could be due to the differences in their phytochemical composition [9]. According to the criteria of classification of the antibacterial activity of the phytochemicals [28], the extracts used in this study were moderately and/or weak active (256 ≤ MIC < 1024 μg/mL). Their direct use in the control of MDR bacterial infections could therefore be of limited importance. None-the-less, the obtained results can be considered as interesting when considering the fact that the extracts are obtained directly from edible plant materials.

Efflux pumps are responsible for the reduction of intracellular concentration of antibacterial compounds [29]. To tackle problems related to this phenomenon, an intensive search of efflux pumps inhibitors (EPI) is welcome [30]. The EPI blocks the efflux pumps and leads to the increase of the intracellular concentration of active principle contents of the extracts [29,31]. The activity of *B. oleacera* var*. butyris* extract against the tested bacteria in the presence of PAβN, increased in 75% of the cases. This suggests that some compounds present in this extract could be substrates of efflux pumps [31,32].

The extracts of *B. oleacera* var*. butyris*, *B. oleracea* var*. Italica*, *Basilicum polystachyon* and *C. frutescens* showed significant synergistic effects (0.031 < FIC < 0.5) with the majority of the tested antibiotics against the studied MDR strains. This suggests that the extracts might contain bioactive compounds that, combined with antibiotics, acted at different sites by various mechanisms [33,34]. These data indicate that a combination of these extracts with antibiotics could be envisaged to fight MDR bacteria.

## Conclusion

These results provide promising baseline information for the potential use of *Capsicum frutescens, Brassica oleacera* var*. italica, Basilicum polystachyon* and *Brassica oleacera* var*. butyris*, independently or in combination with some commonly used antibiotics in the fight against MDR Gram-negative bacteria.

## Competing interests

The authors declare that they have no competing interests.

## Authors’ contributions

FTK carried out the study; VK designed the experiments. FTK, AJS, AGF and VK wrote the manuscript; VK, JAKN and DED supervised the work; VK provided the bacterial strains; all authors read and approved the final manuscript.

## Pre-publication history

The pre-publication history for this paper can be accessed here:

http://www.biomedcentral.com/1472-6882/14/258/prepub
